# Distribution and quality of emergency obstetric care service delivery in the Democratic Republic of the Congo: it is time to improve regulatory mechanisms

**DOI:** 10.1186/s12978-019-0772-z

**Published:** 2019-07-15

**Authors:** Dieudonné Mpunga Mukendi, Faustin Chenge, Mala A. Mapatano, Bart Criel, Gilbert Wembodinga

**Affiliations:** 1 Kinshasa School of Public Health, University of Kinshasa, P.O. Box: 11850, Kinshasa I, Kinshasa, Democratic Republic of the Congo; 2 Lubumbashi School of Public Health, University of Lubumbashi, Lubumbashi, Democratic Republic of the Congo; 3 Institute of Tropical Medicine, Antwerpern, Belgium; 4 Centre de connaissances santé en RDC (CCSC), Kinshasa, Democratic Republic of the Congo

**Keywords:** Distribution, Quality, Emergency obstetric care, DRC, Distribution, Qualité, Soins obstétricaux d’urgence, RDC

## Abstract

**Background:**

The Demographic and Health Survey 2013–14 indicated that the Democratic Republic of the Congo (DRC) is still challenged by high maternal and neonatal mortality. The aim of this study was to assess the availability, quality and equity of emergency obstetric care (EmOC) in the DRC.

**Methods:**

A cross-sectional survey of 1,568 health facilities selected by multistage random sampling in 11 provinces of the DRC was conducted in 2014. Data were collected through interviews, document reviews, and direct observation of service delivery. Collected data included availability, quality, and equity of EmOC depending on the location (urban vs. rural), administrative identity, type of facility, and province. Associations between variables were tested by Pearson’s chi-squared test using an alpha significance level of 0.05.

**Results:**

A total of 1,555 health facilities (99.2%) were surveyed. Of these, 9.1% provided basic EmOC and 2.9% provided comprehensive EmOC. The care was unequally distributed across the provinces and urban vs. rural areas; it was more available in urban areas, with the provinces of Kinshasa and Nord-Kivu being favored compared to other provinces. Caesarean section and blood transfusions were provided by health centers (6.5 and 9.0%, respectively) and health posts (2.3 and 2.3%, respectively), despite current guidelines disallowing the practice. None of the facilities provided quality EmOC, mainly due to the lack of proper standards and guidelines.

**Conclusions:**

The distribution and quality of EmOC are problematic. The lack of regulation and monitoring appears to be a key contributing factor. We recommend the Ministry of Health go beyond merely granting funds, and also ensure the establishment and monitoring of appropriate standard operating procedures for providers.

## Résumé

**Contexte:**

Selon les résultats de l’Enquête Démographique et de Santé 2013–2014, la mortalité maternelle et néonatale reste encore élevée en République Démocratique du Congo (RDC). L’objectif de cette étude était d’évaluer la disponibilité, la qualité et l’équité des soins obstétricaux d’urgence (SOU) en RDC.

**Méthodes:**

En 2014, une enquête transversale était organisée dans 11 provinces de la RDC sur un échantillon de 1 568 formations sanitaires (FOSA) sélectionnées par échantillonnage aléatoire à plusieurs degrés. Les données étaient collectées à travers les interviews, la revue documentaire et l’observation directe. Les informations collectées ont porté sur la disponibilité, la qualité et l’équité des SOU en fonction de la localisation de la FOSA (urbaine ou rurale), son appartenance administrative (publique ou privée), le type de FOSA et la province. Les associations entre les variables étaient mesurées par le test du chi carré de Pearson, au seuil de signification alpha de 0,05.

**Résultats:**

Au total, 1 555 FOSA (99,2%) étaient enquêtées. Parmi celles-ci, 9,1% fournissaient les SOU de base et 2,9% les SOU complets. L’offre des SOU était inégalement répartie à travers les provinces et selon la localisation de la FOSA, le milieu urbain étant plus favorisé. Les provinces de Kinshasa et du Nord-Kivu étaient mieux servies par rapport aux autres provinces. La césarienne et la transfusion sanguine étaient aussi fournis par les centres de santé (6,5% et 9,0% respectivement) et par les postes de santé (2,3% et 2,3% respectivement) et ce, malgré que les normes d’organisation des zones de santé ne le permet pas. Aucune FOSA ne fournissait les SOU de qualité, suite à l’indisponibilité des normes et directives appropriées.

**Conclusions:**

La distribution et la qualité des SOU sont encore problématiques. La faible réglementation et la faible supervision des FOSA semble en être un des facteurs contributifs. Nous recommandons au ministère de la santé d’aller au-delà du simple octroi de fonds et d’assurer la mise en place et le suivi des procédures et standards appropriés pour la prestation de service.

## Plain English summary

In the Democratic Republic of the Congo (DRC), mothers and newborns continue to die from preventable causes, especially at delivery. The aim of this study was to assess the availability, quality, and equity of emergency obstetric care during childbirth in the DRC. A survey was conducted in 2014 to collect information on 1,555 of 1,568 randomly selected health facilities in 11 provinces of the DRC through interviews with health providers, document reviews, and direct observation of service delivery. We found that emergency childbirth care was unequally distributed across the country based on the province and the location of the facility. Care was more available in urban areas, particularly the provinces of Kinshasa and Nord-Kivu. We found that caesarean section and blood transfusions were provided by health centers and health posts, which goes against national policy. None of the facilities provided high quality emergency childbirth care, mainly due to a lack of proper standards and guidelines. Thus, the distribution and quality of emergency obstetric care remains problematic in the DRC, with a lack of regulation as a key contributing factor. We recommend the Ministry of Health ensure the establishment and monitoring of appropriate standard operating procedures for providers.

## Background

Maternal mortality remains high worldwide, despite a 44% decline between 1990 and 2015. Low-income countries in Africa and Asia carry the greatest burden [1]. Since 1990, a 53% decrease in global under-five mortality has been recorded, but neonatal mortality has declined slower than post-neonatal under-five mortality [2], accounting for 45% of all deaths before 5 years [3].

The Democratic Republic of the Congo (DRC) faces the challenge of low public funding for the health sector. This low-income country did not reach the targets set for Millennium Development Goals 4 and 5 [4, 5]. Despite the high rate of vaginal delivery in health facilities hiding significant disparities between provinces, the maternal mortality rate is high, estimated at 846 deaths per 100,000 live births, with neonatal mortality of 28 deaths per 1000 live births. Maternal deaths accounts for 35% of all deaths among women aged 15–49 years [6].

Most maternal deaths are due to preventable causes, such as hemorrhage, hypertensive disorders, and sepsis, which contribute to more than half of maternal deaths and near-misses [7, 8]. On the other hand, acute intrapartum emergencies and poor fetal oxygenation commonly contribute to stillbirth and neonatal deaths [9–12]. The risk factors for maternal and perinatal mortality are strongly entangled, with the first 24 h after childbirth being the riskiest period [13, 14]. Three-quarters of maternal deaths could be prevented if health facilities provide a package of high-quality maternal care [15]. The low availability and accessibility to health services, long distance from households, particularly in rural areas, poorly trained staff (doctors and nurses), insufficient materials and equipment, and deficits in monitoring of service delivery are some of the reasons for the persistently high number of death among women and children in low income countries [5, 16, 17]. Emergency obstetric care (EmOC) has been shown to be effective in reducing mortality among mothers and newborns [18, 19]. Basic EmOC includes seven basic interventions: assisted vaginal delivery, removal of retained products of conception, manual removal of the placenta, basic neonatal resuscitation, and parenteral administration of oxytocin, antibiotics, and anticonvulsants. Comprehensive EmOC also includes blood transfusion and cesarean section [20]. Increasing prompt obstetric interventions, such as assisted delivery (cesarean section and induction of labor), contributes to decreased stillbirth rates [21] and prevents maternal, neonatal, and infant mortality associated with obstetric emergencies [8, 22, 23]. According to the WHO [24], to achieve the targets of the Sustainable Development Goals (SDGs) by 2030, the coverage and utilization of evidence-based interventions need to be improved. The quality of EmOC can be measured in many ways, including utilization of Donabedian’s model of quality medical care, which is based on three elements: structure of health care in terms of input, material, staff, funds, and organizational structure; processes used to deliver care (i.e., respect of standards of care); and outcomes [25].

The availability of EmOC is still scarce in the DRC [16, 26, 27]. A study conducted in nine hospitals in 2007 showed that none met the criteria for a basic or comprehensive EmOC facility [26]. In 2012, the availability of EmOC was estimated at 6% in three provinces in the DRC, higher in public facilities than in private or church-owned facilities [16]. Forty percent of health facilities applied active management during the third stage of labor (AMTSL) and used the partogram, with provincial disparities ​​ranging from 16 to 76% [28]. At the time of this study, no nation-level investigation had yet been conducted on the availability, quality, and equity of EmOC. The objective of the present study was to assess the availability and quality of EmOC and its equity in the DRC.

## Methods

### Study setting

The DRC extends over a large land mass the size of Western Europe. The country faces several major challenges in the EmOC needs of its population due, at least in part, to poor supply chain management, which greatly hinders the delivery of any type of health service. Despite the persistence of these challenges, reproductive health services are well attended by women aged 15–49 years; an estimated 88% of pregnant women benefit from antenatal care (ANC) and 80% of deliveries occur in health facilities. However, modern contraceptive prevalence remains low, estimated at 8% and the fertility rate is still high at 6.6 children per woman [6].

The health system is organized at the national, provincial, and local level. All health facilities have to provide reproductive health services, such as ANC, deliveries, postnatal care (PNC), family planning, and post-abortion care. The Health District consists of two types of health facilities: first line health centers and a district referral hospital. Based on the DRC’s policy documents, the former provide primary health care, including basic EmOC, whereas the latter provides specialized care, which includes comprehensive EmOC, imagery, and laboratory services. Two other ‘non-compulsory’ types of facilities exist: health posts and referral health centers, which deliver services that are not clearly defined [29, 30]. The health sector is characterized by public underfunding, the uncontrolled production of doctors and nurses, in association with the under-production of qualified midwives. The health infrastructure is insufficient and outdated, and the functioning of health facilities is essentially ensured by patients’ payments.

### Study design

This is a cross-sectional study conducted in health facilities in the DRC from April 2014 to June 2014. Four types of facilities were included in the study: hospitals, referral health centers, health centers, and health posts [30]. To be eligible for the study, each facility had to be listed on the Ministry of Health’s roster of facilities and to have provided data to the National Health Information System (NHIS) in the 6 months prior to the study. Before selecting health facilities, the researchers and health officials reviewed the list from each province and updated the roster of facilities reporting to the NHIS. The sampling frame contained only functional facilities. For this study, we used 11 strata, equivalent to the 11 provinces of the DRC. Four substrata corresponded to each type of health facility. To calculate the sample size for each substratum, we used a proportion of 0.5 of facilities that were supposed to have the characteristic of interest, given that the proportion of facilities providing EmOC was unknown. Systematic random sampling, using a sampling interval, helped select the visited health facilities in each substratum. Of the 15,998 functioning health facilities, this procedure yielded a sample of 1,568 facilities.

### Main variables of the study

An index of availability of EmOC and an index of quality of EmOC were calculated by modifying WHO-proposed tools [31]. The index of availability of EmOC was based on four criteria a facility had to meet to be considered as offering EmOC: infrastructure, a specific room dedicated to assisted vaginal delivery (a delivery room); a staff member is assigned to reproductive health activities, such as assisted vaginal delivery, and family planning; at the time of the survey or over a period of 6 months before the study, the facility offers seven functions defined as basic EmOC (assisted vaginal delivery, removal of retained products of conception, manual removal of the placenta, basic neonatal resuscitation, and parenteral administration of oxytocin, antibiotics, and anticonvulsants) or nine functions defined for comprehensive EmOC (all basic care plus blood transfusion and caesarean section) [20]; and evidence of service utilization based on service statistics (e.g., at least one assisted delivery recorded during the 6 months preceding the survey).

The quality index for basic EmOC was based on four elements: the presence of at least one staff member trained in EmOC during the 2 years preceding the survey; existence of basic EmOC delivery guideline documents; availability of material and equipment, including delivery kits, birthing bed, partograph, examination light, manual vacuum, sterilization equipment, ambulance for emergency transport, suction apparatus, manual vacuum extraction, ball and face mask; and availability of drugs and products, such as gloves, disinfectant, injectable uterotonics, injectable antibiotics, infusion solutions, ophthalmic antibiotic ointment, and magnesium sulfate.

The quality index for comprehensive EmOC was based on the following five elements: availability of at least one staff member trained in each category of care (comprehensive EmOC, surgery, anesthesia) in the prior 2 years; existence of comprehensive EmOC delivery guideline documents; availability of material and equipment, including a baby incubator and anesthesia equipment; availability of drugs and products, such as sufficient blood supply, secure blood supply, lidocaine 5%, epinephrine (injection), halothane (inhalation), atropine (injection), thiopental (powder), suxamethonium bromide (powder), and ketamine (injection); and diagnostic capability, such as blood grouping test and cross-compatibility test.

These elements focus mostly on Donabedian’s first dimension of quality care, concentrating on the structure of care. Donabedian’s second dimension, process of care, was captured in the researchers’ index by observing whether EmOC service delivery guidelines exist and are used. As the elements included in our index comprise a modest measure of quality, only facilities that met all of the above criteria were classified as having high quality; if one or more of the criteria were not met, the facility was assessed as having low quality EmOC. In order to determine the efforts needed to improve the quality, particularly when it was poor, we calculated the index ‘operational capacity of EmOC’, which indicates the proportion of quality elements available in health facilities and, indirectly, those to be provided.

Independent variables included the administrative identity of health facilities, location, types of health facilities, and provinces.

### Data collection procedure

Before collecting the data, we contacted provincial health officials to determine how to access each selected facility and what resources were needed. At each facility, data were collected by two staff members (doctors and nurses) recruited from health facilities not selected for the study and trained as interviewers. They visited all facilities and collected data through structured interviews with managers and heads of reproductive health services, performed document reviews, and made direct observations. One interviewer asked the questions and recorded the answers on a paper form while another recorded the same information on a laptop computer. Before leaving the facility, the two interviewers resolved any discrepancies between the paper and electronic forms. Quality control was done by supervisors who revisited 10% of facilities selected randomly to validate the data.

### Data analysis

All data were weighted by stratum before analysis. Microsoft Excel 2010 was used to produce graphs and charts and WINPEPI version 11.54 for analysis and testing of associations. The indices of availability and quality of EmOC were calculated as a proportion of all facilities according to the independent variables mentioned above. Pearson’s chi-squared test was used to test the association of different variables. All hypotheses were tested using an alpha significance level of 0.05.

### Ethical review

This study was reviewed and approved by the Ethics (Human Subjects) National Committee (approbation number 07/CNES/BN/PMMF/2013). The research team obtained authorizations from national and provincial health authorities prior to the survey. Data were collected anonymously after obtaining informed consent from the facilities.

## Results

The researchers successfully surveyed 1,555 of the 1,568 health facilities included in the sample (99.2%). The 13 facilities for which data were not collected were extremely difficult to access geographically.

### Availability of EmOC signal functions

**Fig. 1:**
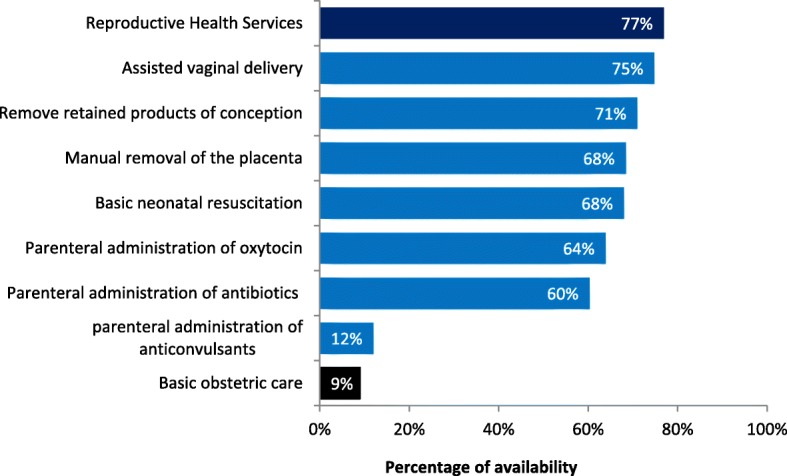
Percentage of facilities with reproductive health services and those with specific interventions for basic obstetric and neonatal care services, as defined by the Availability Index, DRC, 2014 (*N* = 1,555)

Of the 1555 facilities surveyed, 76.9% offered reproductive health services (Fig. 1). Assisted vaginal delivery was the most commonly available (74.8%) of the seven basic EmOC signal functions, followed by removal of retained products of conception (71.0%), manual removal of the placenta (68.4%), and basic neonatal resuscitation (68.0%). Parenteral administration of anticonvulsants was the least available (12.0%). Of all health facilities, 9.1% offered basic EmOC as defined by the index of availability.

**Fig. 2:**
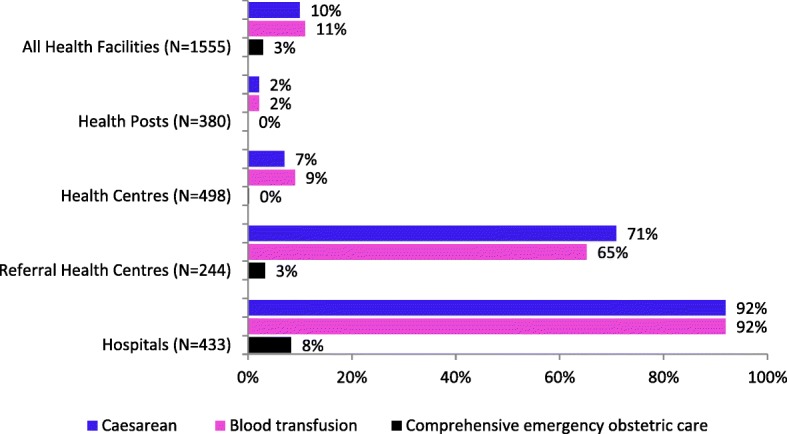
Percentage of health facilities with comprehensive emergency obstetric care, by type of facility, as defined by the Availability Index, DRC, 2014 (*N* = 1,555)

Caesarean sections and blood transfusions were most often offered in hospitals (91.8 and 91.7%, respectively; Fig. 2), followed by the referral health centers (71.3 and 65.0%, respectively). Some referral health centers that performed caesarean sections did not provide blood transfusions. Cesarean sections and blood transfusions were provided in a few health centers (6.5 and 9.0%, respectively) and health posts (2.3 and 2.3%, respectively). Comprehensive EmOC was offered by 8.3% of hospitals, 3.4% of referral health centers, 0.2% of health centers, and none of the health posts, representing 3.0% of health facilities providing cesarean section (Fig. 2).

Of the 1,555 health facilities visited, 9.1% offered basic EmOC and 2.9% offered comprehensive EmOC (Table 1). Basic EmOC was 2-times more available than comprehensive EmOC (*p* < 0.001). The availability of basic and comprehensive EmOC was higher in hospitals (17.8 and 9.1%, respectively), but decreased to its lowest level in the health posts (1.3 and 0.0%, respectively).

**Table 1:** Percentage of health facilities with EmOC according to select characteristics

	No. Of Health Facilities	Basic EmOC Available		Comprehensive EmOC Available	
		n (%)	*P* value	n (%)	*P* value
Total	1555	142 (9.1)		45 (2.9)	
Type			< 0.001		< 0.001
Hospital	433	77 (17.8)		36 (8.3)	
Referral health center	244	45 (9.0)		8 (3.4)	
Health center	498	15 (6.1)		1 (0.2)	
Health post	380	5 (1.3)		0 (0.0)	
Administrative identity			< 0.001		0.012
Public	872	60 (6.9)		17 (2.0)	
Private	683	82 (12.0)		28 (4.3)	
Location			< 0.001		< 0.001
Urban	367	62 (16.9)		24 (7.0)	
Rural	1188	80 (6.7)		21 (1.8)	
Province			< 0.001		
Kinshasa	147	28 (19.0)		11 (7.5)	
Nord Kivu	148	27 (18.2)		11 (7.4)	
Maniema	106	14 (13.2)		4 (3.8)	
Kasaï Oriental	150	19 (12.7)		1 (0.7)	
Equateur	114	13 (11.4)		3 (2.6)	
Katanga	162	11 (6.8)		4 (2.5)	
Sud Kivu	137	9 (6.6)		8 (5.8)	
Kasaï Occidental	139	8 (5.8)		1 (0.7)	
Kongo Central	161	6 (3.7)		3 (1.9)	
Province Orientale	151	5 (3.3)		1 (0.7)	
Bandundu	140	2 (1.4)		1 (0.7)	

The availability of basic and comprehensive EmOC services was significantly higher in private health facilities than in public health facilities, and higher in urban area than in rural areas (p < 0.001). In addition, at a province level, basic EmOC was more available than comprehensive EmOC. Basic EmOC was most widely available in the provinces of Kinshasa and Nord Kivu, and infrequently available in Bandundu and Province Orientale; 4 out of 11 provinces (Kasai Oriental, Kasai Occidental, Province Orientale, and Bandundu) had almost non-existent comprehensive EmOC (< 1% of facilities) (Table 1).

### Quality of EmOC signal functions among health facilities with reproductive health services

**Fig. 3:**
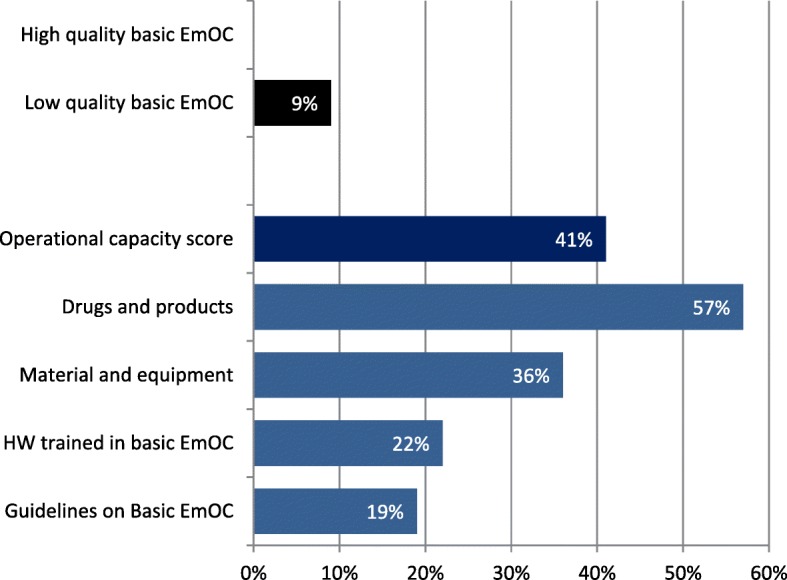
Percentage of facilities with Reproductive Health Services that were rated high quality for basic emergency obstetric care quality, DRC, 2014 (*N* = 1196 Health Facilities)

None of the health facilities offering reproductive health services provided high quality basic EmOC according to the calculated index of quality (Fig. 3). Of the four quality criteria assessed, the availability of drugs and products (57%) was highest, followed by the availability of material and equipment (36%) and health workers trained in basic EmOC (22%). Guidelines on basic EmOC was the least available criterion. A total of 19 quality elements were evaluated, of which the ball and face mask (4%), manual vacuum extraction (6%), magnesium sulfate injection (10%), and suction apparatus (10%) were the least commonly available (data not shown). Health facilities had, on average, 8 of 19 elements; the operational capacity of basic EmOC was estimated to be 41% (Fig. 3).

**Fig. 4:**
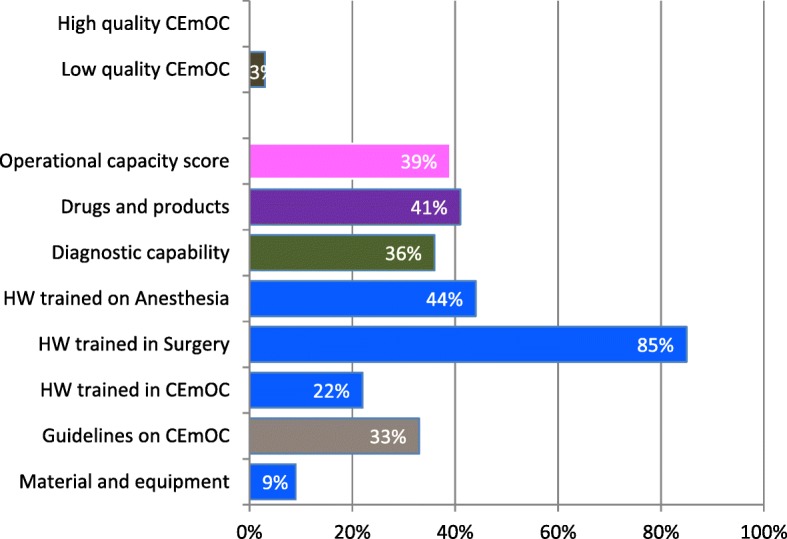
Percentage of health facilities with comprehensive emergency obstetric care available that were rated “high” on service quality, DRC, 2014 (*N* = 1196 Health Facilities)

As with basic EmOC, none of the surveyed health facilities offered high quality comprehensive EmOC. Of the 647 health facilities offering at least one comprehensive EmOC signal function, less than half had drugs and products (41%), the needed diagnostic capability (36%) and availability of guidelines on comprehensive EmOC (33%); 85% of facilities had health workers trained in surgery, 44% in anesthesia and 22% in comprehensive EmOC. Material and equipment requested for service delivery were the least available of all criteria (9%). Of the 17 quality elements for comprehensive EmOC, the least available were anesthesia equipment (3%), suxamethonium bromide powder (12%), cross-compatibility test (14%), thiopental powder (15%), and baby incubators (15%) (data not shown). The health facilities had, on average, 7 of 17 quality elements; the operational capacity of comprehensive EmOC was estimated to be 39% (Fig. 4).

As none of the health facilities offered high quality basic or comprehensive EmOC in this study, Tables 2 and 3 describe the operational capacity by type of health facility, location, administrative identity, and province. The operational capacity was calculated for facilities that offered one or more EmOC signal function, as highlighted in Fig. 1. The operational capacity for basic EmOC was highest in Kinshasa, Nord Kivu, Sud Kivu, Kongo Central, and Katanga, and was low in the remaining provinces. The operational capacity decreased when moving from hospitals to health posts, and was higher in private health facilities and urban areas. Trained health workers, guidelines, and ambulance services were the three lowest quality elements of basic EmOC, despite differences between provinces, types of facilities, administrative identity, and location (Table 2).

**Table 2:** Operational capacity of health facilities to provide quality basic EmOC according to select characteristics (*N* = 1,196)

	No. Facilities Offering RHS	Guidelines On Basic EmOC, n (%)	*P* value	HWw Trained In Basic EmOC, n (%)	*P* value	Ambulance, n (%)	*P* value	Operational Capacity Score, %^a^
Province			< 0.001		< 0.001		< 0.001	
Kinshasa	91	35 (38.5)		42 (46.2)		16 (21.3)		60.1
Nord Kivu	124	27 (21.8)		35 (28.2)		20 (20.3)		57.3
Sud Kivu	106	37 (34.9)		37 (34.9)		30 (35.7)		54.8
Kongo Central	126	22 (17.5)		14 (11.1)		42 (41.3)		46.5
Katanga	128	15 (11.7)		13 (10.2)		9 (8.8)		42.2
Bandundu	112	21 (18.8)		26 (23.2)		2 (2.0)		40.8
Kasaï Occidental	109	26 (23.9)		31 (28.4)		19 (22.0)		40.6
Province Orientale	99	8 (8.1)		18 (18.2)		7 (8.6)		39.7
Kasaï Oriental	126	13 (10.3)		6 (4.8)		1 (0.8)		33.4
Equateur	91	3 (3.3)		27 (29.7)		2 (2.6)		31.6
Maniema	84	18 (21.4)		16 (19.0)		0 (0.0)		29.2
Type			< 0.001		< 0.001		< 0.001	
Hospital	432	117 (27.1)		136 (31.5)		83 (19.2)		68.5
Referral health center	195	47 (24.1)		45 (23.1)		26 (13.3)		56.8
Health center	396	49 (12.4)		76 (19.2)		28 (7.1)		42.7
Health post	173	12 (6.9)		8 (4.6)		11 (6.4)		29.9
Administrative identity			0.100		0.003		< 0.001	
Public	691	119 (17.2)		132 (19.1)		63 (9.1)		38.3
Private	505	106 (20.9)		133 (26.3)		85 (16.8)		46.6
Location			0.006		0.002		< 0.001	
Urban	269	66 (24.5)		78 (29.0)		54 (20.1)		51.8
Rural	927	159 (17.2)		187 (20.2)		94 (10.1)		38.9
Total	1196	225 (18.8)		265 (22.1)		147 (12.3)		41.3

*RHS* reproductive health services (76.9% of facilities in this study offered RHS), *HW* healthcare worker

^a^When no health facilities offered quality basic or comprehensive EmOC, the operational capacity was calculated as a proportion of the corresponding items available at each health facility

**Table 3:** Operational capacity of health facilities to provide quality comprehensive EmOC according to select characteristics (*N* = 647)

	No. Of Facilities	Guidelines On Comprehensive EmOC, n (%)	P Value	HWs Trained In CEmOC, n (%)	P value	Operational Capacity Score, %^a^
Province			< 0.001		< 0.001	
Sud Kivu	60	32 (53.3)		34 (56.7)		53.5
Katanga	78	20 (25.6)		14 (17.9)		51.3
Nord Kivu	82	47 (57.3)		48 (58.5)		46.7
Kinshasa	66	33 (50.0)		34 (51.5)		43.4
Kongo Central	73	34 (46.6)		29 (39.7)		41.4
Bandundu	60	10 (16.7)		13 (21.7)		34.6
Equateur	36	5 (13.9)		6 (16.7)		34.6
Maniema	38	7 (18.4)		10 (26.3)		34.6
Kasaï Occidental	50	11 (22.0)		25 (50.0)		34.4
Province Orientale	57	10 (17.5)		18 (31.6)		32.9
Kasai Oriental	47	7 (14.9)		4 (8.5)		20.4
Type			–		–	
Hospital	433	155 (35.8)		167 (38.6)		47.4
Referral health center	174	55 (31.6)		56 (32.2)		38.5
Health center	35	5 (14.3)		11 (31.4)		36.5
Health post	5	1 (17.1)		1 (17.1)		11.9
Administrative identity			0.211		0.110	
Public	316	98 (31.0)		105 (33.2)		34.8
Private	331	118 (35.6)		130 (39.2)		41.9
Location			0.003		0.001	
Urban	185	78 (42.2)		86 (46.5)		47.8
Rural	462	138 (29.9)		149 (32.3)		34.3
Total	647	216 (33.4)		235 (36.3)		38.7

*HW* healthcare worker

^a^When no health facilities offered quality basic or comprehensive EmOC, the operational capacity was calculated as a proportion of the corresponding items available at each health facility

Of the 17 comprehensive EmOC quality elements, trained health workers and guidelines were the least available. The provinces of Sud Kivu and Katanga had the highest operational capacity, whereas Province Orientale and Kasai Oriental had the lowest. Operational capacity was also higher in hospitals, private facilities, and urban health facilities. A significant difference was found between provinces (*p* < 0.001), facility location (*p* = 0.003), and the availability of guidelines. Moreover, a significant difference was found between provinces (p < 0.001), facility location (*p* = 0.001), and trained health workers for comprehensive EmOC (Table 3).

## Discussion

The results of this study are of great importance in improving the organization of the health system in the DRC. Despite the high percentage (76.9%) of health facilities offering reproductive health services nationally, only 9.1% provide basic EmOC and 2.9% comprehensive EmOC, and none of the EmOC services are of high quality.

Delivery is part of the minimum package of activities for health centers in the DRC [29] and should be provided by all facilities integrated into the primary health care strategy. This study illustrates that a quarter of all health facilities do not provide assisted vaginal delivery. The low availability and limited geographical accessibility of health services, particularly in rural areas, are indirect causes of the persistently higher maternal and neonatal mortality [13]. Despite the high availability of assisted vaginal delivery, basic EmOC was offered in less than 10% of facilities. These results confirm the findings of contemporary studies in the DRC that have reported low availability of EmOC [16, 26, 27]. One of these studies also indicated the long distance from households to health facilities as a key barrier to utilization of health services [16]. The researchers found that a third of health facilities did not offer any of the basic EmOC signal functions; parenteral administration of anticonvulsants, oxytocin, and antibiotics were the least available. These findings confirm those of a 2012 survey [28] in which the above elements were also lacking, and corroborate the poor management of hemorrhage, eclampsia, and bacterial infection highlighted in the majority of developing countries [7]. AMTSL would make it possible to effectively address postpartum hemorrhages if practiced by each provider during each delivery. The low availability of parenteral administration of oxytocin after delivery, associated with the low availability of the partograph and an insufficient number of trained health workers reflects weak application of AMTSL. These results are consistent with those found in a mapping interventions in maternal, neonatal, and infant health [28] and a study focused on EmOC organized in three provinces of the DRC [16]. The poor availability of oxytocin, anticonvulsants, and antibiotics may contribute to the persistently high rate of maternal deaths.

Cesarean sections were more often provided in hospitals and referral health centers. Few surveyed health facilities provided comprehensive EmOC. Some did not offer cesarean section or blood transfusion services. Available data indicate that caesarean delivery rates in African and Asian countries are < 3%, and are particularly low in rural areas, below the WHO recommended threshold of 5–15%. A caesarean delivery rate < 5% is indicative of the lack of access to EmOC, human resource issues, and other health system challenges [20, 32]. It also suggests unmet needs.

Assessments of the national needs in EmOC have shown that met needs remain low in nine surveyed African countries (28.0%), suggesting that many women who suffer from obstetric complications are not treated appropriately [20]. According to the results of this study, none of the health facilities offered quality basic or comprehensive EmOC. To ensure the quality of care, health services have to provide protection and safety to both the mother and newborn through the availability of nine signal functions of EmOC provided by trained health professionals [20]. The poor quality of care highlighted in this study indicates a higher risk of morbidity and death. The quality of EmOC was poor, mainly due to the lack of qualified human resources (doctors and nurses) and the low availability and use of guidelines and material. Over the past 10 years, the DRC has experienced a decline in under-five mortality, which remains less pronounced in newborns [6]. Our findings indirectly reflect the poor quality of neonatal care, with low availability of critical elements for basic neonatal resuscitation.

Problems in equity of access to health services were identified in this study. Six of 11 provinces (Katanga, Sud-Kivu, Kasai-Occidental, Bandundu, Kongo Central, and Province Orientale) had few health facilities with EmOC signal functions. These results corroborate the findings of a study that focused on family planning services in the DRC, which highlighted problems of equity [33]. Private health facilities offered better basic and comprehensive EmOC than public facilities. These results challenge the effectiveness of the control mechanisms introduced by health officials in the public health sector. Public facilities do not seem to be held accountable for better organization of health care services offered to the community.

The findings of this study reflect the weak regulation or standardization of the health system. For example, basic EmOC signal functions are unevenly distributed across the provinces; they are more available in urban than rural areas. These results raise the problem of equity, which can be linked to both financial and geographic access to health services, as reported in other studies [31, 34, 35]. Moreover, both basic and comprehensive EmOC were less available in health centers compared to hospitals, and hospitals are often difficult to access geographically, especially in rural areas, accentuating the inequalities [29, 30]. In many health systems, limited incentives exist for providers in rural and poor areas. The financial support from government and other partners often stops in urban areas or certain privileged rural areas [5].

The poor regulatory power of health officials in the DRC is reflected in another result from this study – referral health centers and health posts, two ‘facultative’ facilities in a health district, still exist, as the health system strengthening strategy suggesting their transformation into health centers is not well implemented. In addition, the entire package of activities that can be offered by these two health facilities is not clearly defined [30].

Caesarean section should be provided by hospitals and, in a few situations, by referral health centers. In this study, some hospitals and referral health centers did not provide these services because of human resource, material, and equipment constraints. On the other hand, 6.5% of health centers and 2.3% of health posts practiced caesarean sections and blood transfusions without the necessary skills, qualified human resources, and equipment. Some of them practiced caesarean section while being unable to provide a blood transfusion in an emergency. In the DRC, health centers and health posts are primarily called on to offer first-level care and to refer patients to hospitals for specialized care, such as surgery, if necessary. One of the reasons for this situation is that certain hospitals and referral health centers that are supposed to provide cesarean section and blood transfusion do not offer such services, and this leaves room for opportunistic health facilities. Furthermore, underfunding of health facilities by public funds pushes managers to base operating costs on patient payments. The total health expenditure in the DRC was estimated to be 21.03 USD/inhabitant/year. Unfortunately, approximately 42% of this comes from out-of-pocket payments, maintaining the spectrum of poverty and increasing vulnerability [36].

In addition, the functioning of health facilities is not regularly or appropriately monitored by district-level health officials due to, among other things, underfunding and demotivation, creating a vicious circle. The WHO and others describe this deregulation as being due mainly to underfunding [37, 38]. The regulatory power of the Ministry of Health seems to be ineffectual in many low income countries [38, 39], making health systems inefficient and expensive, accentuating inequalities, and leading to low quality, dangerous, and harmful health care [37, 38].

The WHO calls on countries to adapt current standards and guidelines on EmOC to the local context [40]. The standards for maternal and infant health services formulated by the Ministry of Health in 2012 are not clear; the DRC has not yet set up its own standards and guidelines on EmOC availability and quality [41]. A study conducted in 2011 in the city of Lubumbashi reported that only one health facility provided comprehensive EmOC for a catchment area of 918,819 inhabitants [42], which confirms the low availability of EmOC.

In our study, standards and guidelines for maternal and child health were available in 19.0% of facilities. However, even when available, they were not translated into operational instructions, particularly for the providers at the lowest level of the health system. When applied, operational instructions can help improve the quality of EmOC. One of the regulatory tools, the health district organizational standards developed in the DRC, is already outdated; in 2006, it was not envisaged to assign doctors to health centers. At the time of this study, some of these structures were managed by doctors with a lack of qualified midwives. Moreover, with the presence of a physician, almost all health facilities falsely become ‘secondary hospitals’ according to the health workers and community, even if their technical plateau and care package had not improved. The presence of doctors could be an opportunity to improve the quality at the first line of care, if the Ministry of Health is engaged in the supervision and follow-up of these doctors. Currently, the health facilities in urban areas without doctors are likely to be deserted by patients for those with physicians [43]. The presence of physicians at the first line of care is probably largely untapped. An important reason is a lack of vision in the DRC health system to extend the role of general practitioner at the health center level. Another reason is that the guidelines issued by the Ministry of Health, particularly on maternal and child health, are often lacking in facilities and often not contextualized. This situation could lead to poor application of these guidelines by the managers of health facilities and could contribute to the lack of feedback to national health officials, which would have rendered the updating of these documents possible.

### Strengths and limitations

This is the first study to assess the access and quality of EmOC signal functions on a national scale in the DRC. One of the strengths of this study is the use of stratification and systematic sampling strategies to collect data representing the whole country, despite important challenges in this context. The study had a high response rate, and the quality control performed by the supervisors helped eliminate discrepancies in the data. The main limitation is related to the type of sampling that excluded all health facilities not integrated into the NHIS, which could have led to an over-estimation of EmOC. Another limitation may be the possible confusion, despite the training of data collectors, between ‘assisted vaginal delivery’ and ‘vaginal delivery’, as both terms are used indiscriminately in the common language of local providers. The last limitation may be related to the fact that we did not collect data on service utilization or other quality dimensions.

## Conclusions

The availability and quality of reproductive health services in the DRC remain problematic. Health services are inequitably distributed throughout the country, with better availability in urban areas, and with significant differences between provinces. Private health facilities are likely to have better availability of EmOC than public health facilities. The noted poor regulation of the health system seems to be one of the main causes of the current situation. The regulatory role of the Ministry of Health should be strengthened to ensure equity, provide sufficient funds, standards, and guidelines, and control implementation by health providers.

## Data Availability

The datasets used for this study are part of an important database produced to evaluate the DRC’s National Health Development Plan 2011–15. They are available from the corresponding author upon reasonable request.

## Abbreviations

AMTSL: Active management during the third stage of labor
DRC: Democratic Republic of the Congo
EmOC: Emergency obstetric care
HW: Health worker
NHIS: National Health Information System
RHS: Reproductive health service
SDG: Sustainable development goal
WHO: World Health Organization

## References

[1] World Health Organization (WHO), UNICEF, UNFPA, World Bank, United Nations. Trends in Maternal Mortality: 1990 to 2015: Estimates by WHO, UNICEF, UNFPA, World Bank Group and the United Nations population division. Geneva: WHO; 2015. Available: http://www.who.int/reproductivehealth/publications/monitoring/maternal-mortality-2015/en/. Accessed 24 May 2017.

[2] UNICEF, World Health Organization (WHO), World Bank, United Nations (2015). Levels & Trends in Child Mortality. Report 2015. Estimates Developed by the UN Inter-agency Group for Child Mortality Estimation.

[3] Liu L, Hill K, Oza S, Hogan D, Chu Y, Laxminarayan R, Temmerman M, Walker N (2016). Levels and causes of mortality under age five years. Disease control priorities (third edition): volume 2, reproductive, maternal, newborn, and child health, edited by black R.

[4] Ministère de la Santé Publique. Plan National de Développement Sanitaire 2011-2015: République Démocratique du Congo; 2011. Available from: ww.minisanterdc.cd/. Accessed 23 June 2015

[5] Ministère de la Santé Publique. Plan National de Développement Sanitaire de deuxième génération 2016–2020: RDC; 2016. p. 1–100. Available from: http://www.nationalplanningcycles.org/sites/default/files/planning_cycle_repository/democratic_republic_of_congo/pnds_2016-2020_version_finale_29_avril_2016.pdf. Accessed 3 Apr 2017

[6] Ministère du Plan et Suivi de la Mise en Œuvre de la Révolution de la Modernité (MPSMRM); Ministère de la Santé Publique (MSP); ICF International. Enquête démographique et de santé en République Démocratique du Congo 2013–2014. Rockville (MD): ICF International. Co-published by MPSMRM and MSP. 2014. Available from: https://dhsprogram.com/pubs/pdf/FR300/FR300.pdf. Accessed 3 Apr 2017

[7] Say Lale, Chou Doris, Gemmill Alison, Tunçalp Özge, Moller Ann-Beth, Daniels Jane, Gülmezoglu A Metin, Temmerman Marleen, Alkema Leontine (2014). Global causes of maternal death: a WHO systematic analysis. The Lancet Global Health.

[8] Souza JP, Gülmezoglu AM, Vogel J, Carroli G, Lumbiganon P (2013). Moving beyond essential interventions for reduction of maternal mortality (the WHO multi-country survey on maternal and newborn health): a cross-sectional study. Lancet.

[9] Azra Haider B, Bhutta ZA (2006). Birth asphyxia in developing countries: current status and public health implications. Curr Probl Pediatr Adolesc Health Care.

[10] Goldenberg RL, McClure EM, Bann CM (2007). The relationship of intrapartum and antepartum stillbirth rates to measures of obstetric care in developed and developing countries. Acta Obstet Gynecol Scand.

[11] Halloran DR, McClure E, Chakraborty H, Chomba E, Wright LL, Carlo WA (2009). Birth asphyxia survivors in a developing country. J Perinatol.

[12] Lawn J, Shibuya K, Stein C (2005). No cry at birth: global estimates of intrapartum stillbirths and intrapartum-related neonatal deaths. Bull World Health Organ.

[13] Kassebaum NJ, Bertozzi-villa A, Coggeshall MS, Shackelford KA, Steiner C, Heuton KR (2014). Global, regional, and national levels and causes of maternal mortality during 1990–2013: a systematic analysis for the global burden of disease study 2013. Lancet..

[14] Prual A, de Bernis L, El Joud DO (2002). Rôle potentiel de la consultation prénatale dans la lutte contre la mortalité maternelle et la mortalité néonatale en Afrique subsaharienne. Gynecol Obstet Biol Reprod.

[15] Richard F, Witter S, De Brouwere e V (2008). Réduire les barrières financières aux soins obstétricaux dans les pays à faibles ressources, Studies in Health Services Organisation and Policy, 25.

[16] Ministère de la Santé Publique, Fonds des Nations Unies pour la population, Organisation Mondiale de la Santé, Averting Maternal Death and Disability. Soins obstétricaux et néonatals d’urgence dans les structures de soins en RDC: évaluation des besoins dans trois provinces. Rapport d’enquête: RDC; 2012. Available from: http://drc.unfpa.org/sites/default/files/pub-pdf/SONU_RAPPORT_ENQUETE_FINAL_DU_7_12_2012.pdf. Accessed 23 June 2017

[17] Paxton A, Maine D, Freedman L, Fry D, Lobis S (2005). The evidence for emergency obstetric care. Int J Gynecol Obstet.

[18] Lee ACC, Cousens S, Wall SN, Niermeyer S, Darmstadt GL, Carlo WA (2011). Neonatal resuscitation and immediate newborn assessment and stimulation for the prevention of neonatal deaths: a systematic review, meta-analysis and Delphi estimation of mortality effect. BMC Public Health.

[19] World Health Organization (2016). Standards for improving quality of maternal and newborn care in health facilities.

[20] Darmstadt GL, Yakoob MY, Haws RA, Menezes EV, Soomro T, Bhutta ZA (2009). Reducing stillbirths: interventions during labour. BMC Pregnancy Childbirth.

[21] Betran AP, Torloni MR, Zhang J, Ye J, Mikolajczyk R, Deneux-Tharaux C, Oladapo OT, Souza JP, Tunçalp Ö, Vogel JP, Gülmezoglu AM (2015). What Is the Optimal Rate of Caesarean Section at Population Level? A Systematic Review of Ecologic Studies. Reproductive Health.

[22] Li XF, Fortney JA, Kotelchuck M, Glover LH (1996). The postpartum period: the key to maternal mortality. Inter J Gynecol Obst.

[23] Hofmeyr G. Justus, Haws Rachel A., Bergström Staffan, Lee Anne CC, Okong Pius, Darmstadt Gary L., Mullany Luke C., Oo Eh Kalu Shwe, Lawn Joy E. (2009). Obstetric care in low-resource settings: What, who, and how to overcome challenges to scale up?. International Journal of Gynecology & Obstetrics.

[24] USAID. Maternal and Child Health Integrated Program (MCHIP). Essential Obstetric and Neonatal Care: Implementation guide of the program. Jhpiego Corporation. 2012. Available: https://www.mcsprogram.org/wp-content/uploads/2016/02/MCHIP_Moz_FinalReport.pdf. Accessed 12 July 2019.

[25] Donabedian. The quality of medical care: a concept in search of a definition. J Fam Pract 1979, 9, N°2: 227–284. 110905

[26] Casey SE, Mitchell KT, Amisi IM, Haliza MM, Aveledi B, Kalenga P, Austin J (2009). Use of facility assessment data to improve reproductive health service delivery in the Democratic Republic of the Congo. Confl Health.

[27] Casey SE, Chynoweth SK, Cornier N, Gallagher MC, Wheeler EE (2015). Progress and gaps in reproductive health services in three humanitarian settings: mixed-methods case studies. Confl Health.

[28] Ministère de la Santé Publique. Cartographie des interventions et intervenants de la santé de la mère, du nouveau-né et de l’enfant y compris la planification familiale en RD Congo: RDC; 2012. Available from http://familyplanning-drc.net/docs/Rapport%20final%20cartographie%20de%20la%20SMNE%2029%20octobre%202012.pdf. Accessed 3 April 2017

[29] Ministère de la Santé. Normes d’organisation des Zones de santé: RDC; 2006. p. 11–27. Available from: https://docplayer.fr/34761187-Recueil-des-normes-d-organisation-et-de-fonctionnement-des-structures-sanitairess-dela-zone-de-sante-en-republique-democratique-du-congo.html. Accessed 12 Sept 2012.

[30] Ministère de la Santé. Stratégie de renforcement du système de santé. 2nd ed: RDC; 2010. Available from: www.minisanterdc.cd/. Accessed 21 May 2015

[31] OMS (2015). Mesurer la disponibilité et la capacité opérationnelle des services (SARA): Un outil d’évaluation des établissements de santé.

[32] Cavallaro FL, Cresswell JA, França GVA, Victora CG, Barros AJD, Ronsmans C (2013). Trends in Caesarean delivery by country and wealth quintile: cross-sectional surveys in southern Asia and sub-Saharan Africa. Bull World Health Organ.

[33] Mpunga Dieudonné, Lumbayi JP, Dikamba Nelly, Mwembo Albert, Ali Mapatano Mala, Wembodinga Gilbert (2017). Availability and Quality of Family Planning Services in the Democratic Republic of the Congo: High Potential for Improvement. Global Health: Science and Practice.

[34] Indonesia. Resident midwives help avert maternal deaths when financial barriers are removed, Immpact, University of Aberdeen, UK, 2 pp. Available: https://assets.publishing.service.gov.uk/media/57a08bd7ed915d3cfd000f98/IndonesiaFactSheet.pdf. Accessed 12 July 2019.

[35] Ronsmans C, Holtz S, Stanton C (2006). Socio-economic differentials in caesarean rates in developing countries: a retrospective analysis. Lancet..

[36] Ministère de la Santé Publique. Rapport des Comptes Nationaux de la santé 2014. RDC, 2016. Available from: https://www.resilientinstitutionsafrica.org/sites/default/files/files/2017/DRC-Rapport_sur_les_comptes_de_la_sante_RDC_2014.pdf. Accessed 19 Nov 2018

[37] Macintosh M (2007). Planning and market regulation: strengths, weaknesses and interactions in the provision of less inequitable and better quality health care.

[38] Organisation Mondiale de la Santé (2008). Les soins de santé primaires : maintenant plus que jamais.

[39] Ferrinho P, Omar MC, de Jesus Fernandes M, Blaise P, Bugalho AM, Van Lerberghe W (2004). Pilfering for survival: how health workers use access to drugs as a coping strategy. Hum Resour Health.

[40] WHO, UNFPA, Unicef, AMDD. Monitoring emergency obstetric care. A handbook: World Health Organization; 2009. Available: whqlibdoc.who.int/publications/2009/9789241547734_eng.pdf. Accessed 22 July 2017.

[41] Ministère de la Santé de la République Démocratique du Congo. Normes d’organisation des soins maternels, infantiles et adolescents. Volumes N°1–8. Kinshasa, RDC. 2012.

[42] Ntambue AM, Malonga FK, Cowgill KD, Dramaix-Wilmet M, Donnen P (2017). Emergency obstetric and neonatal care availability, use, and quality: a cross sectional study in the city of Lubumbashi, Democratic Republic of the Congo, 2011. BMC Pregnancy Childbirth.

[43] Chenge M, Van der Vennet J, Porignon D, Luboya N, Kabyla I, Criel B (2010). La carte sanitaire de la ville de Lubumbashi, République Démocratique du Congo Partie I : problématique de la couverture sanitaire en milieu urbain congolais. Glob Health Promot.

